# Increasing sustainability in food production by using alternative bait in snow crab (*Chionoecetes opilio*) fishery in the barents sea

**DOI:** 10.1016/j.heliyon.2023.e13820

**Published:** 2023-02-17

**Authors:** Kristine Cerbule, Leif Grimsmo, Bent Herrmann, Eduardo Grimaldo

**Affiliations:** a Department of Fisheries Technology, SINTEF Ocean, Trondheim, Norway; b UiT the Arctic University of Norway, Tromsø, Norway; c DTU Aqua, Technical University of Denmark, Hirtshals, Denmark

**Keywords:** Sustainable food production, Bait, Pot fishery, Catch efficiency, Snow crab

## Abstract

The use of food grade wild-captured species as bait for other fisheries questions the sustainability of food production. In pot fisheries, bait is an important factor determining the effectiveness of the gear. In snow crab (*Chionoecetes opilio*) fishery, the pots are normally baited with squid (*Illex* sp.) and herring (*Clupea harengus*). This fishery uses substantial amounts of bait for each pot deployment, and it constitutes one of the largest expenses for operating the pots along with costs for fuel. Furthermore, reliance on bait that originates from wild-capture fisheries questions economic and environmental sustainability, and involves additional use of fuel for capture and transportation of the bait which increases the carbon footprint of the industry. Therefore, the use of alternative bait sources is needed. One such alternative bait source can be originating from processed by-products from commercial fisheries. However, for the new bait to be acceptable for the fishery, it must provide comparable catch efficiency as the traditional bait. Therefore, this study aimed at comparing the performance of a new experimental bait against the traditionally used squid bait in the Barents Sea snow crab fishery. The results showed no statistically significant difference in catch efficiency of target-sized snow crab. Specifically, a formal uncertainty estimation based on nested bootstrapping showed that there was no significant difference in efficiency between bait types for target-sized individuals for soak times typically employed in the fishery. Thereby this shows a potential to increase sustainability in food production and a positive effect on the size selectivity by additionally demonstrating a reduced capture of undersized individuals.

## 1 Introduction

The continued use of resources that could be used for human consumption in further food production has come under criticism due to its relative inefficiency [1]. Specifically, in fisheries, the criticism of inefficiency is often focused on the use of several wild-captured species as bait for other fisheries instead of direct human consumption [1,2]. Such bait production involves high production costs [[3], [4], [5]], and results in large amounts of post-capture processing waste [6,7]. Additional concerns from using bait from wild-capture fisheries include negative ecological consequences and increased fuel consumption for capture and transportation of the bait [8].

In pot fisheries, bait is a substantial part of determining effectiveness of the gear at attracting and capturing the target species. The mechanism behind the catch process in the pot fishery is utilizing the food-search behaviour of the target species with bait being the source attracting the individuals to approach the gear. When the target individuals encounter the pot, the capture is dependent on them entering the pot following the attraction to the bait source placed in the pot [9,10].

The use of larger bait quantity often affects catch rates by increasing the catches of the target species in the pots [9]. However, bait is one of the most significant costs related to the pot fishery [11], along with fuel expenses required due to long distances [3]. Therefore, fishers are subject to a cost-benefit trade-off when selecting the bait type and its quantity for the fishery [2]. Aspects affecting catchability directly impact the economic output. The choice of bait type depends on the species being targeted. However, there are also various physical properties of the bait and its usage that has to be considered such as bait persistence, rate of diffusion and soak time, all of that can have an effect on the catch effectiveness.

**Fig. 1:**
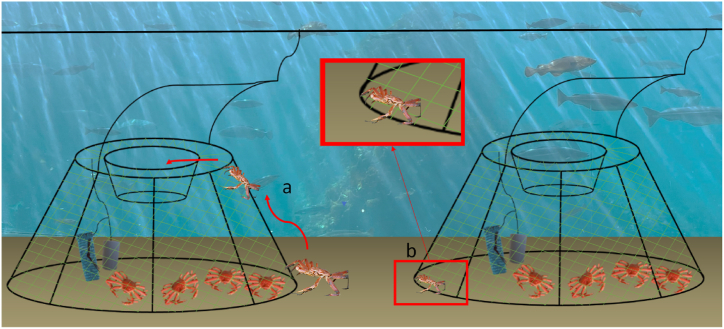
Illustration of the capture process in snow crab pots and the main elements of the gear with (a) showing the entry process of a crab climbing over the pot netting towards the top entrance attracted by bait which is placed in small-meshed bait bags and perforated plastic containers, and (b) the subsequent escaping process of an undersized crab through the netting meshes.

Baited conical pots are the most common gear for targeting snow crab (*Chionoecetes opilio*) in commercial fisheries with the preferred bait being squid (*Illex* sp.) or a combination of squid and herring (*Clupea harengus*) [12]. The capture process of those pots is relying on the crab following the bait odour and climbing to the top entrance of the pot where a circular entrance is located above the bait [13]. After that, the individual falls down into the entrance funnel and is captured by the gear (Fig. 1a). Pots in this fishery are designed to let the undersized crabs (<95 mm CW) escape through the pot meshes once the bait is decayed and they are no longer attracted by the fishing gear (Fig. 1b). However, undersized crabs are captured in some instances, these snow crabs are rapidly released back to the ocean when the pots are recovered. Two bait holders are normally used in the snow crab fishery, a mesh bag and a perforated plastic container (Fig. 1) with the amount of bait ranging from around 700 g–1000 g divided approximately in similar fractions within both bait holders.

The reason for using two bait holders is related to the assumption that bait depletion is longer for bait containers compared to the mesh bags [4]; therefore, functioning for longer pot soaking periods when compared to the bait in the bait bag, which is more rapidly depleted.

In the Barents Sea, the snow crab fishery started up in a small scale in 2012; however it has been expanding with catches reaching 7.428 t (round weight) in 2022 [14]. The regulations in the Norwegian snow crab fishery include a closed season between the 1st of July and the 1st of October, and a maximum of 20% of soft-shell crabs in the catch. In addition, the minimum landing size (MLS) of 95 mm carapace width (CW) is imposed. All undersized individuals must be returned to the sea. Furthermore, each vessel in the snow crab fishery can use up to a maximum of 9000 pots. The number of pots deployed per day can be up to a maximum of 2000 [15]. Bait constitutes a considerable operating cost due to the substantial amount of pots used in the fishery (up to a maximum of 2000 pots per day which equals up to around 2000 kg of bait). For example, in the Barents Sea, most of the fishing vessels use squid bait, and the typical price of 40 NOK/kg gives a bait cost of 20–40 NOK/pot (equals to approximately 2–4 EUR/pot). Thus, there is a need for alternative, effective low-priced, and more sustainable bait. One such bait alternative can involve the by-products from commercial fisheries. Such bait ingredients represent an opportunity to increase sustainability and lower costs by replacing food grade bait from capture fisheries with one that does not offer any nutritional benefit for human consumption. However, for an alternative bait to be used in the fishery, its catch efficiency needs to be comparable to the traditionally used bait type to provide at least the same profitability. In addition, the new bait type should not attract more undersized individuals to the pots since that could considerably increase the sorting time onboard the fishing vessel and expose the small individuals to cold weather conditions that might affect the survival rate after the undersized snow crab are returned to sea.

In the present study, we compared the efficiency of commercially used squid bait and new bait produced from marine by-products from seafood processing. We designed this study to answer the following question: Are there any differences in the catch efficiency of undersized and target-sized snow crab if the conical snow crab pots are baited with new experimental bait compared to traditionally used squid?

## 2 Materials and methods

### 2.1 Sea trials and data collection

Sea trials to test the new bait were conducted in May 2022 onboard the commercial snow crab fishing vessel “Vima” (LOA 69.2 m) at the snow crab fishing grounds in the Barents Sea. The fishing depth was approximately 300 m.

The treatment pots were baited with the new bait produced by Norbait AS (https://www.norbait.com/), Norway. The production of the bait was performed at a processing line developed by Norbait AS 8. April 2022. The main ingredients were the rest raw materials from the production of seafood products including whitefish and squid offal. The control pots were baited with the standard bait of whole frozen squid (*Illex* sp.) delivered by the Norwegian seafood company Domstein Fish AS which is normally used in the commercial snow crab fisheries. During the experiments, both treatment and control pots contained similar amount of bait. Specifically, all treatment and control pots were baited with one small mesh bag and one plastic container placed equally in each pot. Each bait bag and container was baited with approximately 320 g of experimental bait and standard bait for the treatment and control pots, respectively.

**Fig. 2:**
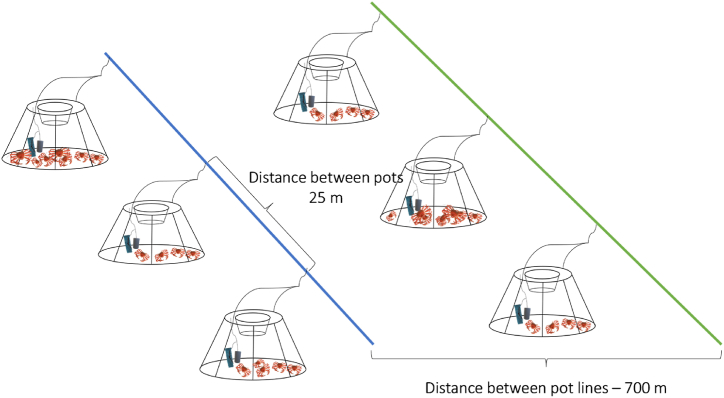
Experimental setup used during the fishing trials. Two mainlines with pots baited with control bait (squid) (blue) and experimental bait (green) were deployed in parallel to each other with 700 m distance between the two. The pots on each mainline had a distance of 25 m. Both mainlines were deployed simultaneously and in the same fishing area. (For interpretation of the references to colour in this figure legend, the reader is referred to the Web version of this article.)

To compare the catch efficiency of pots using the two bait types, the pots of each bait type were deployed on two separate parallel longlines with approximately 700 m distance (Fig. 2). Such distance was chosen to minimise the potential contamination between the two bait types while the pots were deployed. However, the distance between the pot lines (treatment and control group) also could not be extended much more due to potential variations in snow crab abundance.

The distance between the individual pots was 25 m. Both of the parallel longlines were deployed and recovered subsequently; therefore, both lines of pots had similar soaking time. In total, three replicates (experiments) of the parallel lines testing both bait types were deployed during the trials.

After the pots were recovered onboard, the crabs of each pot were sorted into target-sized and undersized individuals and the number of crabs in each fraction was counted for each pot in each line (experimental bait and standard bait) separately. This protocol was followed because of the time constraints and capture rates imposed by sampling during commercial fishing did not enable measuring and registering the precise size for the snow crab captured.

### 2.2 Estimation and comparison of bait capture efficiency

To compare the catch performance between the two bait types, we first estimated the mean number of snow crab captured (expressed as capture per unit effort (CPUE) per deployment) in each pot in treatment pots using the new bait and control pots using the squid bait, separately by:

(1) $$\begin{array}{c} {CPUE}_{uC}=\frac{\sum_{i=1}^{KC}{nuC}_{i}}{KC}\\ \begin{array}{c} {CPUE}_{tC}=\frac{\sum_{i=1}^{KC}{ntC}_{i}}{KC}\\ {CPUE}_{uT}=\frac{\sum_{i=1}^{KT}{nuT}_{i}}{KT} \end{array}\\ {CPUE}_{tT}=\frac{\sum_{i=1}^{KT}{ntT}_{i}}{KT} \end{array}$$

In Eq. (1), *ntC* is the number of target-sized crabs in control pots while *ntT* is the number of target-sized snow crab retained in the treatment pots. Similarly, *nuC* and *nuT* denotes the number of undersized snow crab retained in control and treatment pots, respectively. *KC* and *KT* are the number of pots on control and treatment lines in each experiment, respectively.

The estimation of the mean number of crabs in treatment pot and control pots depends on the spatial and temporal size-dependent availability of snow crab on the fishing ground at the time and location where the experiments are conducted. Therefore, such result provides an estimate that is specific to the snow crab population fished and thus it cannot be extrapolated to other fishing areas and seasons. Further, the absolute catch for each pot type depends on soak time in an unknown way. Specially, the capture process in pots involves an attraction/entry process and a subsequent but overlapping size selection process, where some of the crab that have entered a pot can escape. Both these processes are affected by the intensity of the odour of the specific bait type used, which will change over time. Therefore, assuming, for example, that the total catch increases linearly with increasing soak time is a very questionable assumption to apply. Further, as the capture efficiency may potentially be different for undersized and target-sized snow crab, the estimation of absolute catch efficiency should explicitly include potential size-dependency which again would need detailed information on the availability of undersized and target-sized crab, respectively.

On the contrary, the ratio between the number of crabs captured in treatment (experimental bait) and control pots (squid bait) in each experiment does not require the information about the availability of undersized and target-sized snow crab. This is because they will affect CPUE for the control and treatment groups equally as long as those two groups are fished simultaneously and close to each other such that both groups are exposed to the same density of undersized and target-sized crabs. Therefore, the ratios in CPUE between treatment and control groups are a more robust measure for the effect of bait type than the CPUE for the respective groups. Since this ratio measure does not depend on the snow crab abundancies, these results are more general compared to the CPUE. The ratios in CPUE between treatment and control pots were estimated as follows:

(2) $$\begin{array}{c} {RatioCPUE}_{u}=\frac{{CPUE}_{uT}}{{CPUE}_{uC}}\\ {RatioCPUE}_{t}=\frac{{CPUE}_{tT}}{{CPUE}_{tC}} \end{array}$$

**Fig. 3:**
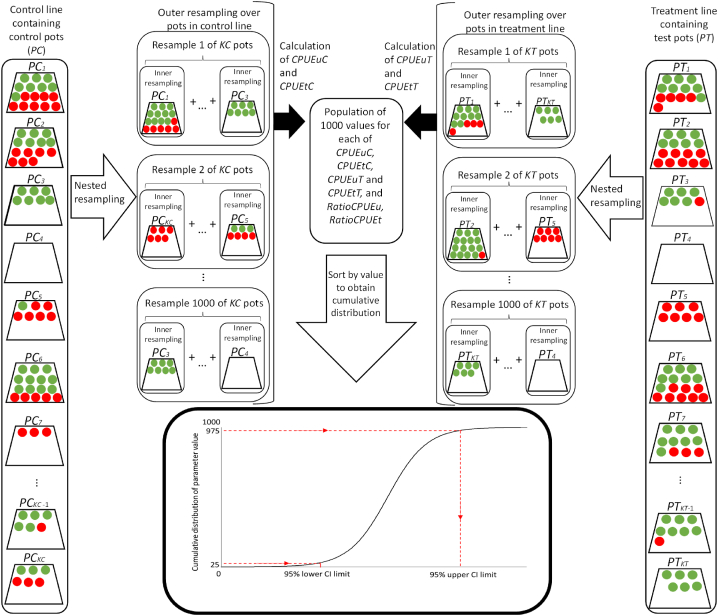
Conceptual diagram with the methodological details of the nested bootstrapping approach applied to estimate uncertainties for each of the performance parameters (CPUEs and *RatioCPUEu* and *RatioCPUEt*). Pots in control line (*PC*_1_–*PC*_*KC*_) and treatment line (*PT*_1_ – *PT*_*KT*_) have different numbers of individuals captured which is represented by green and red circles for target-sized and undersized snow crab, respectively. (For interpretation of the references to colour in this figure legend, the reader is referred to the Web version of this article.)

In Eq. (2), *RatioCPUE*_*u*_ and *RatioCPUE*_*t*_ are ratios between the treatment and control pots for undersized and target-sized snow crab, respectively. Equations (1), (2) are used for each of experiments 1-3 separately to estimate the effect of changing from control bait to treatment bait on snow crab capture efficiency. Uncertainties are obtained using a nested bootstrap approach (Efron, 1982) as described below. First, in an outer resampling loop the groups of control and treatment pots were resampled separately (Fig. 3). Specifically, with *KC* and *KT* pots in the two groups *KC* and *KT* pots, respectively, were drawn with replacement from the two groups. Second, each time a specific pot *i* was drawn in the outer resampling, its catch in terms of number crab *nuC*_*i*_*+ntC*_*i*_ or *nuT*_*i*_*+ntT*_*i*_ was resampled with replacement in an inner resampling between under- and target-sized individuals (Fig. 3). This nested resampling procedure led to a set of values for $\sum_{i=1}^{KC}{nuC}_{i}$, $\sum_{i=1}^{KC}{ntC}_{i}$, $\sum_{i=1}^{KT}{nuT}_{i}$ and $\sum_{i=1}^{KT}{ntT}_{i}$ which by applying (1) led to a set of values for ${CPUE}_{uC}\text{,}$ ${CPUE}_{tC}$, ${CPUE}_{uT}$ and ${CPUE}_{tT}$ (Fig. 3). Finally, using (2) led to values for also ${RatioCPUE}_{u}$ and ${RatioCPUE}_{t}$ (Fig. 3). Repeating this resampling scheme 1000 times led to a population of 1000 results for ${CPUE}_{uC}\text{,}$ ${CPUE}_{tC}$, ${CPUE}_{uT},{CPUE}_{tT}\text{,}$ ${RatioCPUE}_{u}$ and ${RatioCPUE}_{t}$ which were applied to obtain Efron 95% percentile confidence intervals (CI) for each of those performance parameters [16]. Specifically, the 1000 values for each parameter were sorted and ranked after their value. Based on this, the lower bound value for the 95% confidence limit was obtained by inspecting the value for the bootstrap iteration that was the 25th lowest value. Similarly the upper bound confidence limit was the one with the 975th lowest value (Fig. 3). If the CIs for *RatioCPUE*_*u*_ and *RatioCPUE*_*t*_ do not include value 1.0, there is a significant difference between the treatment and control pots for undersized and target-sized snow crab, respectively. This nested bootstrapping approach enables to account for both uncertainty in the performance parameters due to variation in capture rates of snow crab between pots due to local variation in abundancy and in entry probability through the outer resampling loop, and to account for the variation between the capture of under- and target-sizes in the individual pots through the inner resampling loop.

We used the statistical software SELNET for the analysis of the data [17].

## 3 Results

Following the experimental design with control and treatment line, three experiments were conducted separately. Data for each of those three experiments were individually analyzed following the description in section 2.2. Experiments 1, 2 and 3 contained 122, 144 and 186 treatment pots and 246, 260 and 290 control pots where the numbers of target-sized and undersized snow crab were counted (Table 1).In each experiment with a treatment and control group, pots were deployed simultaneously and had the same pot soak time ranging from 9 to 13 days. The number of target- and undersized snow crab in pots using both types of bait varied between the three experiments.

**Table 1:** Experimental data sets of the three experiments. Corresponding start and end position of the lines, depths, soaking time (days), number of treatment and control pots in each experiment, and number of undersized (<95 mm carapace width) and target-sized snow crabs retained in treatment and control pots in each experiment.

Experiment	Position (treatment pots)	Position (control pots)	Distance between pot lines (m)	Date of deployment	Soaking time (days)	Depth (m)	Number of treatment pots (*KC*)	Number of control pots (*KT*)	Number of undersized snow crab in treatment pots (*nuT*)	Number of target-sized snow crab in treatment pots (*ntT*)	Number of undersized snow crab in control pots (*nuC*)	Number of target-sized snow crab in control pots (*nuT*)
1	N75°57–75°58 E32°43-32°55	N75°57–75°56 E32°37-32°54	740	May 05, 2022.	9	310	122	246	40	996	172	2210
2	N76°26–76°26 E33°04–34°15	N76°12–76°18 E33°44-33°58	700	May 18, 2022	9	315	144	260	548	2679	1156	4751
3	N76°07–76°05 E35°16–34°00	N76°18–76°07 E35°07–34°18	700	May 05, 2022	13	300	186	290	910	1585	3238	4249

### 3.1 Capture rates of snow crab in pots with the two bait types

**Fig. 4:**
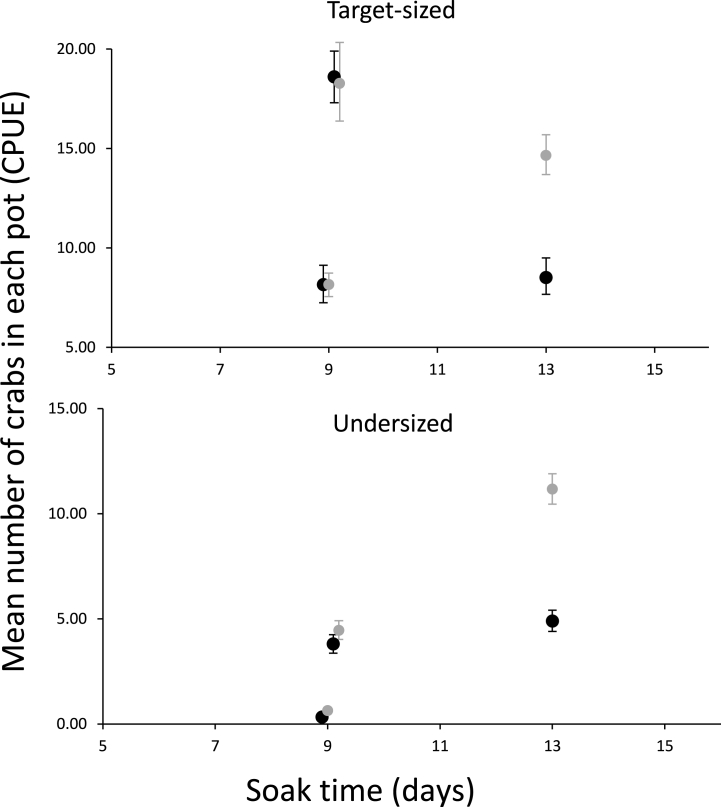
Upper panel: mean number of crabs in treatment (black) and control (grey) pots for target-sized snow crab (≥95 mm CW) (*CPUE*_*tT*_, *CPUE*_*tC*_). Lower panel: mean number of crabs in treatment (black) and control (grey) pots for undersized snow crab (<95 mm CW) (*CPUE*_*uT*_, C*PUE*_*uC*_) captured in each of the three mainlines.

Results in Table 2 and Fig. 4 show that in two of the three experiments there was no significant difference between the mean number of crabs captured in each pot when the two bait types were compared. Specifically, in Experiment 1 and Experiment 2, treatment and control pots captured similar amount of snow crab when using the two different bait types (Fig. 4). The *CPUE* of target-sized crabs were 8 in both treatment (*CPUE*_*tT*_ = 8.16 (CI: 7.25–9.14)) and control bait pots (*CPUE*_*tC*_ = 8.16 (CI: 7.56–8.75)), respectively in Experiment 1. In Experiment 2, both pots also had similar number of target-sized and undersized snow crab (Fig. 4). The mean numbers of captured snow crab varied between the two experiments; however, there were no significant differences between the two experiments (Table 2; Fig. 4).

**Table 2:** Results with the mean number of crabs for target-sized snow crab (≥95 mm CW) and undersized snow crab (<95 mm CW) captured in each of the three mainlines in treatment and control pots. Numbers in parentheses are 95% confidence intervals.

	Experiment 1 (9 days soak time)		Experiment 2 (9 days soak time)		Experiment 3 (13 days soak time)	
	Treatment	Control	Treatment	Control	Treatment	Control
Target (*CPUE*_*t*_)	8.16 (7.25–9.14)	8.15 (7.56–8.75)	18.60 (17.31–19.90)	18.27 (16.38.20.34)	8.52 (7.68–9.50)	14.65 (13.70–15.70)
Undersized (*CPUE*_*u*_)	0.33 (0.19–0.50)	0.63 (0.50.0.77)	3.81 (3.37–4.25)	4.45 (4.02–4.91)	4.89 (4.40–5.41)	11.17 (10.45–11.91)

However, there was a significant difference between the two bait types in Experiment 3, since the control bait pots retained significantly (*CPUE*_*tC*_ = 14.65 (CI: 13.70–15.70)) more crabs compared to the treatment bait (*CPUE*_*tT*_ = 8.52 (CI: 7.68–9.50)) for the target-size snow crab. This difference was also significant for undersized snow crab in the Experiment 3 in the experimental bait pots (*CPUE*_*uT*_ = 4.89 (CI: 4.40–5.41)) and in the control bait pots (*CPUE*_*uC*_ = 11.17 (CI: 10.45–11.91)) (Table 2).

### 3.2 Ratio in capture between the two bait types

**Fig. 5:**
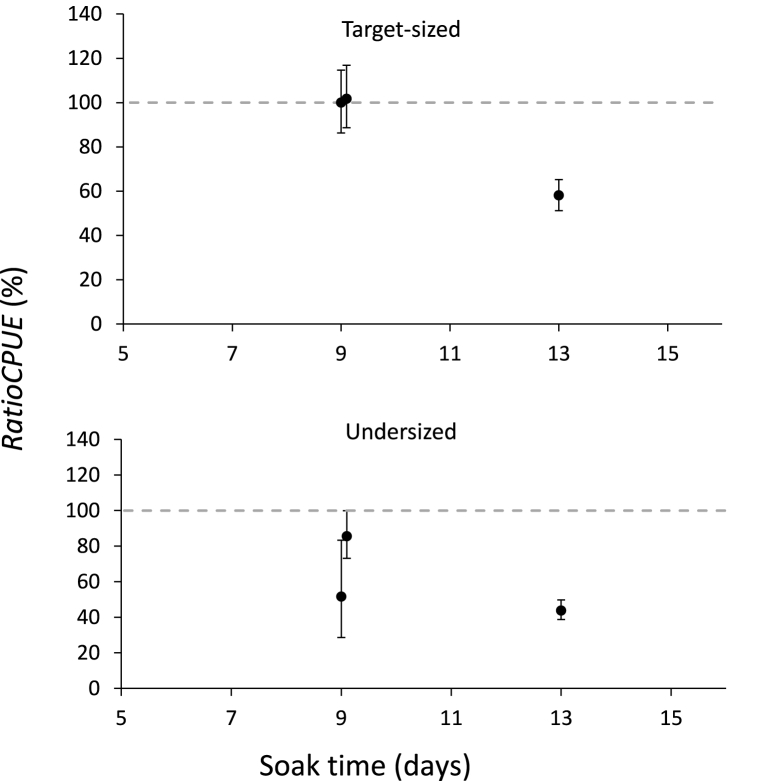
Upper panel: ratios (in %) between treatment and control pots for target-sized snow crab (≥95 mm CW) (*RatioCPUE*_*t*_). Lower panel: ratios (in %) between treatment and control pots for undersized snow crab (<95 mm CW) (*RatioCPUE*_*u*_).

The ratio in capture between pots with experimental and control bait did not show significant differences in two of the three experiments for target-sized snow crab (*RatioCPUE*_*t*_) (Fig. 5; Supplementary material 1). Specifically, the ratio in capture between the treatment and control pots was approximately 100% meaning that the treatment pots capture the same amount of snow crab as the pots using the control bait (i.e., the treatment pots were retaining *RatioCPUE*_*t*_ = 100.11% (CI: 86.26–114.73%) and *RatioCPUE*_*t*_ = 101.81% (CI: 88.68–116.92%) of the target-sized snow crab for Experiment 1 and Experiment 2, respectively (Supplementary material 1)). However, in Experiment 3, the treatment pots captured significantly less snow crab than the pots with the new bait (i.e., *RatioCPUE*_*t*_ = 58.16% (CI: 51.19–65.29%)) of what the control pots were retaining).

Further, the pots using the new bait captured significantly less undersized snow crab in all three experiments (Fig. 5). This difference ranged from *RatioCPUEu* = 43.82% (CI: 38.68–49.77%) in Experiment 3 to *RatioCPUEu* = 85.59% (73.12–99.97%) in Experiment 2 when compared to the corresponding control bait pots (Supplementary material 1).

## 4 Discussion

This study evaluated the performance of a new bait produced from marine by-products against the currently used squid bait in the Barents Sea snow crab fishery. This study aimed at reducing the dependency of using human food resources as bait, and thus increase the sustainability in the food production in the snow crab fishery.

In this study, we observed significant differences in treatment and control pots regarding the lower number of undersized snow crab being retained in the pots using the experimental bait (*RatioCPUE*_*u*_) in all three series. This could be potentially caused by two factors. The first explanation could be that the experimental bait decayed faster compared to the control bait reducing the ability to attract snow crab during the pot deployment. Visual and qualitative observations upon the pot recovery showed that around half of the experimental bait in the bait bag was depleted after 13 days soak time while approximately one fifth was depleted after the short soak time (9 days). However, the experimental bait in the bait container did not decay to the same extent as in the bait bag and was approximately equal to the initial bait amount when the pots where deployed. This was comparable to the squid bait. The second potential explanation is that the experimental bait from the beginning would show a lower attractiveness compared to the traditional squid bait in the control pots. The latter explanation would affect the entrance of target-sized snow crab in a similar way. However, treatment and control pots with short soak times showed no difference in the capture efficiency of target-sized individuals between experimental and control pots. Therefore, we can conclude that a lower initial efficiency of the experimental bait to attract snow crab cannot be a possible explanation. Thus, the results of the observed reduction in undersized snow crab for long soak time pots could indicate that the experimental bait did not maintain its attractiveness as long as the baseline bait (i.e., bait odour decayed faster when compared to the squid bait). However, controlled experiments assessing the efficiency of the experimental bait for different soak times would be needed to assess whether prolonged release of bait odour of experimental bait would increase the catch efficiency of target-sized individuals.

In this study, a large reduction of target-sized snow crab was observed for the experimental pots using a long soak time (13 days). The possible implications of having pots with long soak times is that it allows snow crab to approach the pot from a larger initial distance. In such case, observed lower efficiency of the experimental bait for the long soak time pots would imply a possible lower efficiency of the bait over longer distance. However, this is not relevant for the shorter time since no such reduction was observed for the shorter soaking period (i.e., 9 days soak time). Therefore, while the new bait in this study demonstrated convincing results for a relatively short soak time (9 days) along with some benefits regarding the observed reduction of undersized crab (<95 mm CW), the efficiency with longer soak time where the crab need to be attracted over longer distances can be reduced.

However, the intended soak time in the snow crab fishery is around one week considering the optimal catch efficiency and providing sufficient time for an optimal size selection of undersized snow crab at the seabed [18]. Longer soak times are often associated with operational challenges in the fishery, e.g., related to harsh weather conditions. Therefore, considering this deployment pattern in the fishery, the observed results show an optimal efficiency of the experimental bait. Specifically, for the deployment pattern as intended in the fishery, we obtained an equal catch efficiency of target-sized crab while observing a significantly lower capture of undersized crab. Therefore, in addition to being able to contribute to increasing the sustainability of food production by not using bait that can be used in direct human consumption [1,2, it also has a positive effect on increased release of undersized crab at the seabed. The duration of the bait effect should be adjusted to fishing patterns regarding the optimal soak time for which further experiments would be needed. This process could benefit from a more time-controlled release of the bait molecules [8] by using timed bait release devices, such as the Longsoaker device (Longsoaker Fishing Systems Inc., https://longsoaker.com/).

The results of this study showed that the use of the alternative bait shows similar catch efficiency for target-sized snow crab in the Barents Sea fishery while having a potential under commercial application to reduce the operational costs related to bait used in the fishery. Currently, the production of the experimental bait is done in a small scale as it is still in the development phase and not yet used in the commercial fishery. Therefore, the precise cost difference is unknown; however, the price of the experimental bait should be lower compared to the baseline bait for being accepted commercially. This challenge might be overcome in time with reduction in costs if the production of the experimental bait is scaled up and put in large production. Lower bait costs could allow use larger amount of bait per pot which might increase the catch efficiency of the snow crab pots [9].

From the practical point of view, the use of the new bait would not involve any additional work or resources during the fishing process. Specifically, the storage of the experimental bait is the same as for the squid bait (frozen conditions), and the baiting process is easier due to the consistence of the new bait which does not disintegrate and, therefore, it is easier to apply the same bait amount for each pot. Further, the use of such alternative bait source would contribute to a more sustainable and environmentally friendly fishing practice since the bait is based on by-products from fish processing and would lower the carbon footprint due to lower fuel consumption that otherwise would be used in commercial fishery targeting bait species. Last, and more important yet, the new bait would reduce the dependency of the fishery on using food resources that can be used for direct human consumption.

## Credit author statement

Kristine Cerbule: Conceived and designed the experiments, analyzed and interpreted the data, wrote the paper. Leif Grimsmo: Conceived and designed the experiments, performed the experiments, wrote the paper. Bent Herrmann: Conceived and designed the experiments, analyzed and interpreted the data, contributed reagents, materials, analysis tools or data, wrote the paper. Eduardo Grimaldo: Analyzed and interpreted the data, Wrote the paper.

## Funding statement

This work was supported by the Research Council of Norway: "Norbait Crustacean" (project no. 332265) and "Crab Tech II" (project no. 296046) and the Norwegian Directorate of Fisheries.

**Acknowledgments**

We would like to thank the company Norbait AS for providing the experimental bait for the sea trials of this study. We are grateful to the company Opilio AS and the crew on their vessel "Vima" for their valuable help and cooperation onboard during this experiment. We are grateful to the editor and reviewers for their valuable comments, which we feel have improved our manuscript.

## Data availability statement

Data will be made available on request.

## Declaration of competing interest

The authors declare that they have no known competing financial interests or personal relationships that could have appeared to influence the work reported in this paper.
