# Supplementary material for: Increasing sustainability in food production by using alternative bait in snow crab (Chionoecetes opilio) fishery in the barents sea
Source: Heliyon. 2023 Feb 17;9(3):e13820. doi: 10.1016/j.heliyon.2023.e13820 (PMC9981889; doi:10.1016/j.heliyon.2023.e13820)
Supplement: Multimedia component 1 [file mmc1.docx]

**Supplementary material**

**Supplementary material 1.** Results of the ratios in catch per unit effort (*RatioCPUE* (in %)) between treatment and control pots for target-sized snow crab (≥95 mm CW), undersized snow crab (< 95 mm CW). Numbers in parentheses are 95% confidence intervals.

|  | Experiment 1  (9 days soak time) | Experiment 2  (9 days soak time) | Experiment 3  (13 days soak time) |
| --- | --- | --- | --- |
| Target (*RatioCPUE_t_*) | 100.11 (86.26-114.73) | 101.81 (88.68-116.92) | 58.16 (51.19-65.29) |
| Undersized (*RatioCPUE_u_*) | 51.66 (28.57-83.41) | 85.59 (73.12-99.97) | 43.82 (38.68-49.77) |
